# The Role of Stress, Trauma, and Negative Affect in Alcohol Misuse and Alcohol Use Disorder in Women

**DOI:** 10.35946/arcr.v40.2.05

**Published:** 2020-08-20

**Authors:** Maria Isabel Barros Guinle, Rajita Sinha

**Affiliations:** 1 Yale Stress Center, Yale School of Medicine, New Haven, Connecticut

**Keywords:** girls and women, sex differences, early trauma, child maltreatment, alcohol craving

## Abstract

Recent evidence indicates that the United States is facing a public health crisis of alcohol misuse and alcohol use disorder (AUD), which has been fueled in part by dramatic rises in binge and heavy drinking and prevalence of AUD in women. Historically, alcohol misuse and AUD have been more prevalent in men than in women. However, recent evidence on data from the past decade shows increases in AUD prevalence rates that are associated with substantially higher binge and heavy drinking and AUD prevalence in women compared to men. This paper first addresses the key roles of stress, trauma, childhood maltreatment, negative affect, and mood and anxiety disorders; sex differences in the presentation of these psychosocial and psychological factors; and their contributions to alcohol misuse, escalation to binge and heavy drinking, and transition to AUD in women. Also examined are potential central and peripheral biological mechanisms by which stressors and traumatic experiences, as well as chronic stress states—including depression and anxiety—may facilitate differential pathways to alcohol misuse, escalation, and transition to AUD in women. Finally, this paper discusses major gaps in the literature on sex differences in these areas as well as the need for greater research on sex-specific pathways to alcohol misuse and transition to AUD, so as to support a more comprehensive understanding of AUD etiology and for the development of new strategies for prevention and treatment of alcohol misuse and AUD in women.

## INTRODUCTION

There has been a global increase in alcohol misuse and rates of alcohol use disorder (AUD) over the last two decades.^1^ Recent substantial increases in the United States come from dramatic rises in the prevalence of alcohol misuse and AUD in women relative to men (women, 84% increase; men, 35% increase).^2^ This dramatic rise stems from increases in hazardous and binge drinking in girls during adolescence as well as in women.^3^ Even though alcohol misuse and AUD are more prevalent in men than in women, there are no sex differences in prevalence of alcohol use during adolescence.^4^ These increases are especially alarming given the fact that women tend to experience greater alcohol-related health problems than do men.^5^ This article focuses on the roles of stress, trauma, childhood maltreatment, negative affect, and mood and anxiety disorders and their contributions to the increases in alcohol misuse, escalation of binge and heavy drinking, and transition to AUD in women. Although there are likely additional genetic and social factors and related mechanisms that may contribute to specific risks of binge drinking and AUD in women, a review of this literature is beyond the scope of this review. Rather, this article focuses on the psychosocial and biological processes by which stress, trauma, negative affect, and mood and anxiety disorders increase the risk of binge and heavy drinking, AUD, and relapse.

## PSYCHOSOCIAL FACTORS INVOLVED IN THE ONSET AND PREVALENCE OF AUD IN WOMEN

Women in the United States are largely overrepresented in stress-related psychopathology rates,^6^ and stress along with drug-related environmental cues are among the most important risk factors driving alcohol seeking, maintenance, and relapse.^7^ Studies suggest that men and women differ in risk trajectories for the development of AUD and in AUD-related health consequences.^8^ For example, women are more likely than men to experience certain types of stressors, such as sexual trauma,^9^ and higher levels of stress have been shown to increase alcohol misuse and AUD vulnerability.^10^ Also, women demonstrate a significantly “more rapid and risk-oriented path to compulsive drug seeking,”^11^ pointing to a significant need to understand sex differences in risk for AUD development and maintenance in order to develop novel prevention and treatment approaches for AUD in women.

### Psychosocial Factors of Early Trauma, Maltreatment, and Adversity

Early trauma, maltreatment, and cumulative adversity are psychosocial stress factors that have long been associated with alcohol misuse, development of AUD, AUD maintenance, and relapse.^10^ Both boys and girls face physical and emotional abuse and neglect, sexual abuse, and cumulative adversity stemming from specific adverse childhood experiences such as substance use and mental health problems in the home, parental discord, and divorce, which are each associated with greater alcohol initiation in childhood.^12^ However, girls and women face significantly higher rates of childhood sexual abuse and violent victimization.^13^ Notably, higher rates of sexual abuse and violent victimization, especially in girls and women, are factors that produce the highest odds ratios for association with heavy drinking, drinking to cope with negative affect, and development of AUD.^10^,^12^,^14^

### Sex Differences in Stress Factors, Early Onset Alcohol Misuse, and AUD

An extensive number of studies point to a positive association between negative affect, trauma, adversity, and chronic stress and vulnerability in developing AUD. Recent studies have shown that girls who report a history of abuse before adulthood are more vulnerable to developing AUD.^15^ Other studies have found that adolescents who face a number of negative life events show increased levels of drug use (and misuse) compared to those who do not face these adverse events.^7^,^10^ Exposure to early life stress may be especially harmful for women, who are exposed to more high-impact trauma (e.g., sexual abuse) than men are, and at a younger age.^16^ Thus, early trauma and chronic adversity both may increase vulnerability to alcohol use initiation, as well as maintenance, especially in girls. However, it is important to consider estimation biases, as women may be more likely to endorse stressful life events; thus, the contribution of these factors to binge drinking and AUD risk among women may be influenced by such estimation biases.

A study by Cheng and Anthony conducted between 2006 and 2014 assessed the dates of first full drink and first heavy drinking episode in around 33,000 females and males (ages 12 to 21) in the United States who had their first heavy drinking episode within the past 24 months.^15^ Their findings revealed that, among adolescents who started to drink between ages 11 and 14, females progressed to a heavy drinking episode more quickly than males. This suggests that when drinking starts before age 15, females are at greater risk than males of progressing to a heavy drinking episode. When considered with the information that girls are more likely than boys to suffer sexual abuse before age 18, these findings raise the possibility that sexual abuse and other trauma, and victimization-related increases may contribute to increased risk of alcohol misuse and development of AUD in women.^17^ However, the specific contribution of these factors to the development of AUD in women needs to be further explored.

## PSYCHOLOGICAL ASPECTS OF STRESS AND TRAUMA EFFECTS ON AUD IN WOMEN

Experiencing stress, trauma, and adversity activates psychological processes of cognitive, affective, and behavioral emotion regulation and self-control to cope with and adapt to such negative life circumstances. During adolescence and young adulthood, emotion regulation becomes particularly relevant because of the rapid brain changes in regions associated with regulating emotion, stress, reward, and higher-order cognitive functioning; such changes underlie the significant biological and psychological changes that boys and girls undergo throughout adolescent development.^18^ Alcohol experimentation occurs frequently during adolescence and young adulthood, and there is a higher risk for the development of AUD or substance use disorder during this time.^19^ Findings indicate that exposure to early trauma and life stressors is associated with greater difficulties in emotional experiences, behavioral control, executive function, and decision-making, which contribute to behavioral control of alcohol intake, and thus could be one pathway that contributes to early onset of alcohol intake and risk of alcohol and substance use disorders.^12^,^19^ Discussed below are the sex differences and impact of negative affect, mood and anxiety symptoms, and post-traumatic stress disorder (PTSD) and their contribution to development of binge and heavy drinking and AUD in women.

### Negative Affect and Alcohol Intake

**Figure 1:**
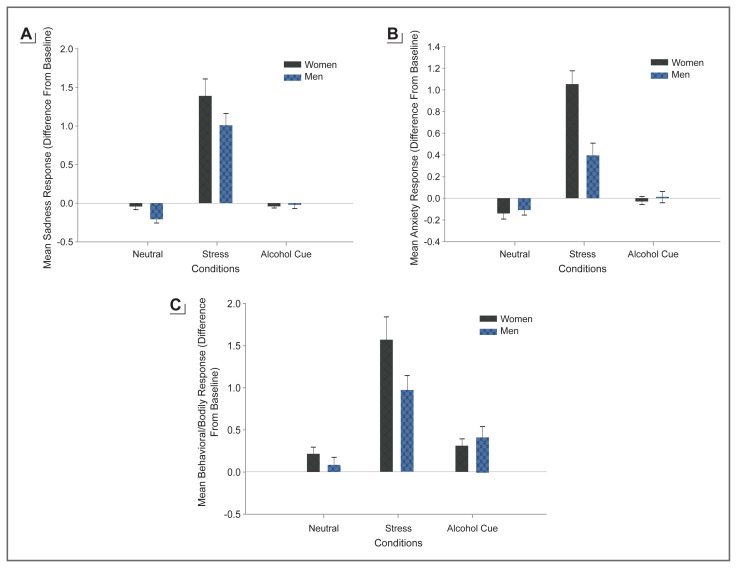
Gender differences in socially drinking volunteers’ average subjective responses to individually calibrated exposure to stress, alcohol cue, and neutral-relaxing control provocation conditions, assessed repeatedly over time in an experimental study. Figure 1a: Average subjective sadness response over time to neutral, stress, and alcohol cue conditions by gender (in stress: women > men, *p* = .01). Figure 1b: Average subjective anxiety response over time to neutral, stress, and alcohol cue conditions by gender (in stress: women > men, *p* < .0001). Figure 1c: Average observed nonverbal behavioral and body responses to neutral, stress, and alcohol cue conditions by gender (in stress: women > men, *p* = .04). *Source:* Reproduced with permission from Chaplin et al. 2008.^22^ Copyright © 2008 Research Society on Alcoholism and the International Society for Biomedical Research on Alcoholism. Published by Wiley-Blackwell. All rights reserved.

Negative affect is broadly defined as a state of emotional distress, and is associated with unpleasant feelings, such as anxiety, fear, anger, irritability, and sadness. Repeated and cumulative exposure to stress, trauma, adversity, and maltreatment is associated with greater levels of negative affect, anxiety, and depressed mood. Past literature suggests that women report more negative affect compared to men,^20^ and higher negative affect has been linked to greater emotion dysregulation and associated with affective, anxiety, and substance use disorders.^10^,^21^ A previous experimental study exposed healthy social drinkers to emotional stress, alcohol cues, and a control neutral relaxing cue using a personalized guided imagery method that individually calibrates stress imagery so as to remove any provocation-related bias between men and women.^22^ Results indicated that men and women were similar in cue-induced craving ratings. However, women reported greater stress-provoked sadness, anxiety, and body sensations compared to men (see Figure 1). These data indicate sex differences in stress and negative affect responses in women versus men, separate from alcohol motivation.

Higher levels of negative affect have specifically been linked to initiation and relapse in alcohol and other substance use disorders.^23^ In adolescents, negative affect is strongly associated with the onset of drinking and alcohol misuse, and higher levels of negative affect are also associated with greater child maltreatment, victimization, and adversity.^23^ Girls show greater negative affect such as sadness in response to early life stress than boys,^19^ similar to findings for adults (and as shown in Figure 1). A number of studies have shown that emotional stress and negative affect also elicit significant alcohol craving,^10^ and negative affect and anxiety are key symptoms of alcohol withdrawal that are further exacerbated by exposure to alcohol cues.^7^ Such a link between stress and negative affect and alcohol motivation highlights the need to assess sex differences and women-specific vulnerability in processes underlying the association between stress and negative affect and alcohol intake, alcohol misuse, and risk of AUD.

Negative affect becomes an important component in the development of AUD in women because past literature has documented that, while men tend to consume alcohol to enhance positive feelings,^24^ women more frequently consume alcohol in response to negative emotions.^11^,^25^ Much like the association between early trauma and substance use, negative affect, such as temperamental negative mood, has also been associated with the development and maintenance of substance use disorders.^11^ Negative emotions, drinking to regulate negative affect, and stress are among the factors associated with increasing rates of AUD in women.^11^ Furthermore, studies have also shown that, in addition to trauma, abuse, and chronic stress, negative affect is predictive of alcohol misuse and addiction vulnerability.^10^ Thus, temperamental negative emotionality, which is often documented as higher in women and is linked to substance use vulnerability, may place women at a higher risk of subsequent alcohol and substance misuse, but its specific role in women’s substance misuse needs further investigation.

### Sex Differences in Anxiety and Depression

Gender gaps in rates of mental illnesses tend to emerge and/or widen during puberty and have been associated with the rise of different sex steroid hormones in boys and girls that occurs during this period. Before puberty, boys and girls have similar rates of depression; however, soon after puberty, depression becomes twice as prevalent in girls than in boys until late adulthood.^26^ This is also true of other mental conditions such as anxiety disorders.^18^ Adult women report more mental health problems than men,^21^ with women with AUD reporting greater mental health problems than women without AUD. In fact, affective disorders have been shown to be the most commonly comorbid psychiatric disorders in individuals with substance use disorder, including AUD.^10^ Even though there exists a representation and estimation bias of women in epidemiological mental health studies, a better understanding of sex-based differences in mental health is crucial to understanding specific risk factors in the development of AUD in women.

Stress is significantly associated with affective and anxiety disorders, raising the issue of whether these disorders contribute to the association between stress and AUD.^11^ Research has shown that individuals with anxiety disorders who reported drinking to cope with their anxiety symptoms drank more alcohol and had a higher rate of DSM-IV alcohol dependence than those who did not report drinking to lessen their symptoms.^27^ There are higher rates of AUD in those with PTSD than in those without PTSD,^28^ and PTSD precedes AUD more often in women than in men.^29^ Both stress and trauma exposure experimentally increase alcohol craving,^30^ and women with both PTSD and AUD report higher levels of trauma, anxiety, and mood symptoms than men.^31^ Furthermore, studies have found that co-occurring AUD, mood and anxiety disorders, and PTSD are associated with higher relapse rates than AUD without such comorbidity.^32^,^33^ Women present different biological, psychological, and physiological effects of alcohol misuse that are crucial to the maintenance of their alcohol use.^5^,^11^ For this reason, sex differences in mental health not only are relevant in the development of AUD, but also need further consideration, especially with regard to prognosis and treatment outcome. Due to the differential physiological and subjective effects of alcohol use in women,^5^ AUD symptoms and progression of disease are accelerated in women, including progression to comorbidities of AUD with other psychopathology such as depression, phobias, and other anxiety and affective illnesses.^11^,^21^

## BIOLOGICAL FACTORS INVOLVED IN THE ONSET AND PREVALENCE OF AUD IN WOMEN

Exposure to stressful and traumatic events as well as chronic adverse environments trigger a biological stress response characterized by neural, physiological (autonomic), hormonal (hypothalamic-pituitary-adrenal [HPA] axis), and immune response changes to support resilient, adaptive coping.^10^ However, uncontrollable events, repeated or chronic stress, and trauma disrupt these responses, thereby breaking down the adaptive nature of stress responses.^10^ This results in allostasis and maladaptive psychological and behavioral responses that put an individual at risk for neuropsychiatric illnesses, including AUD.^10^ Well-documented sex differences start in childhood and continue throughout the life span in these physiological, hormonal, and immune responses, and in the disruption and adaptations that occur as a result of childhood trauma, chronic adversity, and repeated stress experiences.^10^,^11^,^21^ Findings from the authors of this paper and other studies have shown that repeated stress and childhood trauma result in sex-specific adaptations in the autonomic, HPA axis, and immune responses, which have not been well addressed in the literature on risk of AUD.^10^,^11^ For example, girls and women with childhood maltreatment show a blunted HPA axis stress response,^10^ but those without trauma histories and with high negative affect and mood disorders have a hyperreactive HPA axis response to stress.^10^ Changes such as a hyporeactive HPA axis response to acute stress are associated with greater risk of alcohol misuse and AUD, as documented in large longitudinal studies tracking adolescents through young adulthood.^14^ Thus, these youth may seek out substances to normalize their lower basal level of arousal.

Other studies document the highly sexually dimorphic stress response, represented by girls and women showing a higher autonomic, catecholaminergic, and immune response to stress, whereas boys and men show greater glucocorticoid and HPA axis responses to acute stress.^11^ Recent findings also document that increased exposure to childhood victimization results in higher C-reactive protein levels in girls but not boys,^34^ suggesting more stress-related immune compromise and susceptibility in girls relative to boys. In addition, the HPA axis and the autonomic pathways—including the sympathetic and parasympathetic components that coordinate the peripheral biological stress response—show significant dysregulation associated with early life trauma as well as childhood maltreatment, with sex differences in the extent and nature of dysregulation.^10^,^35^ However, specific data on sex differences are not entirely clear. Chronic stress and comorbid mood and anxiety disorders are also associated with altered stress responses,^21^ with higher stress responses in women with mood disorders and without childhood maltreatment, but also blunted stress responses in women who misuse alcohol or who have AUD.^11^,^36^ These findings highlight that a critical aspect of the biological stress response is the associated plasticity in peripheral and central stress biology associated with repeated stress, trauma, and adversity. The sex-specific nature of the stress response also results in sex-specific adaptations and allostatic responses to repeated or chronic stress, adversity, and early life trauma and maltreatment.^35^ The effects on alcohol motivation and intake of such changes in the stress response are discussed below.

### Alcohol Effects on Stress, Negative Affect, and Motivation for Drinking

**Figure 2:**
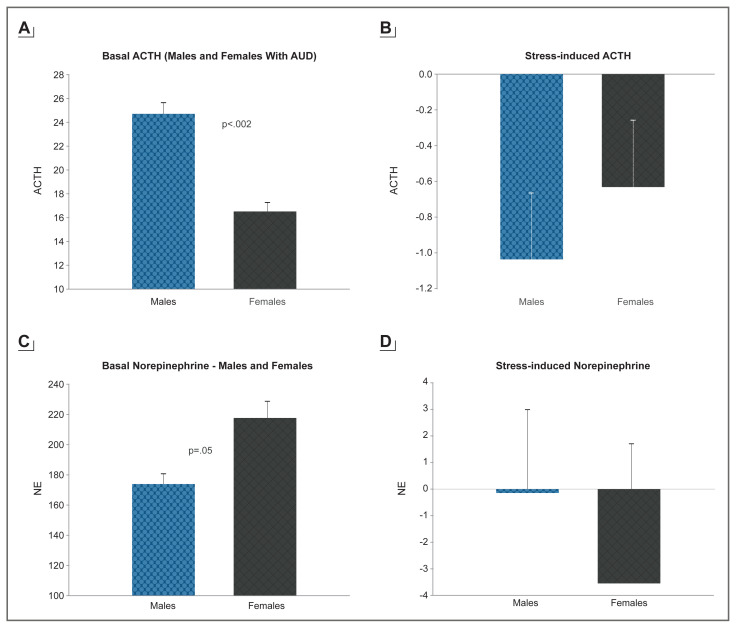
Gender differences in ACTH and NE in men and women with alcohol use disorder (AUD) participating in a laboratory experiment with exposure to individually calibrated stress, alcohol cue, and neutral relaxing imagery on 3 separate days, one condition per day. Figure 2a and Figure 2b: ACTH differences between males and females with AUD at baseline (a) and following stress exposure (b) relative to their neutral response. Attenuation of the diurnal drop is shown in females (Stress > Neutral, *p* = .0009) but not in males. Figure 2c and Figure 2d: NE differences between males and females with AUD at baseline (a) and following stress exposure (b) relative to their neutral response. Attenuation of the diurnal drop is shown in males, but not in females (Neutral > Stress, *p* < .0001). *Note:* ACTH, adrenocorticotropic hormone; NE, norepinephrine. All rights reserved.

Alcohol consumption dramatically affects human physiology, and repeated high-intensity use and misuse is associated with significant neuroadaptations and breakdown of the brain and peripheral systems that coordinate stress, emotion, and reward regulation.^36^ Growing evidence suggests that these adaptations promote a feedforward development of compulsive motivation for alcohol use and misuse.^10^,^21^,^33^ Not only does alcohol stimulate striatal dopaminergic pathways, but it also directly stimulates the HPA axis and affects glucocorticoid receptors in extrahypothalamic, limbic, forebrain, and medial prefrontal cortex (mPFC) circuits associated with the development and progression of AUD.^36^ Alcohol-associated neuroadaptations in HPA axis responses to stress and alcohol cues may serve as psychobiological markers of the cycle of recurring alcohol consumption.^36^ Sex differences in individuals with AUD in the phasic response to stress and in basal tonic levels of HPA axis and the peripheral catecholamines have also been documented.^11^ For example, women with AUD show lower tonic adrenocorticotropic hormone (ACTH) levels but higher norepinephrine (NE) levels relative to men, but also higher relative stress-induced ACTH response and more blunted stress-induced NE response relative to men^11^ (see Figure 2). Thus, neuroadaptations resulting from alcohol consumption (acute and chronic) may facilitate the risk for AUD susceptibility and maintenance in a sex-specific manner.

Following acute, moderate exposure to alcohol or stress, dopaminergic, hypothalamic autonomic, and catecholaminergic pathways have the opportunity to return to their basal states after activation. With alcohol misuse, binge or heavy drinking, and chronic alcohol use, large-scale adaptations and allostatic overload to neuroendocrine regulation circuits occur. These physiological changes have been associated with the transition from controlled to compulsive alcohol seeking in humans.^36^ In fact, in binge and heavy drinkers, a neuroendocrine tolerance to stress and alcohol consumption is observed. For example, a blunted cortisol response to alcohol is observed among individuals with a history of binge or heavy drinking relative to moderate drinkers.^37^ This blunted response to alcohol in those with a history of binge or heavy drinking is identified as neuroendocrine tolerance. Recent findings indicate that, in binge or heavy drinkers, blunted cortisol responses and higher subjective craving are each associated with greater amounts of alcohol intake in the laboratory.^37^ It is important to note that the sample had a majority of men, and sex differences in these effects have yet to be explored. Thus, although binge and heavy alcohol use and associated adaptations in stress biology appear to be involved in the development of neuroendocrine tolerance and in the resulting increases in compulsive motivation,^36^,^37^ neither sex differences in the alcohol-related neuroendocrine tolerance nor the possible sex differences on its effects on alcohol motivation and intake have been explored thus far.

### Alcohol and Stress Interactions on Peripheral and Central Nervous System Responses and Sex Differences

Sex differences have been found in pharmacokinetics and pharmacodynamics of alcohol^38^ as well as in neuroanatomy and chemistry.^24^ Blood alcohol levels rise faster and stay elevated for longer in women than in men. Sex hormones affect the neural pathways and influence neurotransmitter activity, which affects an individual’s physiological and behavioral responses to drugs.^24^ For example, even though men show stronger activation of the brain reward system in response to alcohol than do women,^24^ the female brain suffers more damage and inflammation from alcohol withdrawal.^39^ Important to the current discussion, alcohol stimulates the biological stress pathways in similar ways to psychological stress and trauma.^36^ Similarly, significant adaptations and changes occur as a function of repeated and binge alcohol use in these biological stress pathways, and stress and alcohol misuse may act synergistically to modify HPA as well as autonomic and neural responses to stress and alcohol, which may in turn drive greater craving and compulsive seeking for alcohol.^10^,^36^

A number of studies have linked greater stress reactivity in plasma/salivary cortisol responses as a risk factor for comorbidity of mood disorders and AUD.^40^ Research has also shown that blunted salivary cortisol response to stress is a risk factor for AUD development in at-risk children with a family history of substance misuse or substance use disorder.^41^ There also may be significant variation in these responses as assessed by concentrations in plasma/serum for ACTH, plasma/serum and saliva for cortisol, salivary alpha-amylase (a measure of autonomic adrenergic arousal), and physiological assessments of heart rate and heart rate variability, as a function of extent of chronic stress or trauma exposure.^10^,^42^ Specifically, one study evaluated at-risk prepubertal boys (ages 10 to 12) with fathers with substance use disorder and found that high-risk boys secreted significantly less salivary cortisol in response to an anticipated stressor compared to controls.^41^ These findings were corroborated by another study using a stress task in adolescents, which documented that blunted physiological and emotional responses to stress in adolescents were related to greater risk of alcohol and substance use.^43^ In a larger cohort that also evaluated sex differences in adolescents ages 14 to 17 who were prenatally exposed to cocaine relative to nonexposed youth, elevated basal salivary concentrations of cortisol were found in the at-risk group relative to nonexposed youth.^44^ In contrast, at-risk youth exhibited a blunted salivary cortisol response to a social stressor compared to controls.^44^ Furthermore, sex differences were found in prediction of future substance use: for girls, self-reported sadness in response to the social stressor predicted future drug use, whereas for boys, blunted salivary alpha-amylase (an autonomic nervous system measure) in response to the same social stressor predicted future drug use.^44^ These results suggest that distinct physiological and emotional stress responses among boys and girls are associated with different risk profiles for future drug use.

In another series of studies, impaired neuroendocrine responses to alcohol and to stress have also been associated with an increased motivation for binge or heavy drinking, thereby serving as a potential risk marker for the progression from heavy drinking to DSM-IV alcohol dependence.^45^ In a large population-based study where children were followed longitudinally between ages 14 and 20, the age at which the first alcoholic drink was consumed varied as a function of cortisol levels, and blunted cortisol responses to stress were associated with greater risk of alcohol misuse.^46^ Furthermore, among heavy- and light-drinking adults who were exposed to an oral alcohol challenge and followed for 6 years, heavy drinkers showed greater sensitivity to stimulating effects and lower sensitivity to the sedative effects of alcohol compared to light drinkers.^45^ Moreover, heavy drinkers demonstrated lower salivary cortisol release in response to the alcohol challenge and, 6 years later, presented with a greater number of AUD symptoms than did light drinkers.^45^ These findings suggest that alcohol and stress significantly impact the psychological and biological stress responses—altering affect, mood, and anxiety as well as biological stress responses. However, a significant gap remains in understanding sex differences in these effects given that differences by gender have not been well studied in the literature.

One of the effects of acute administration of alcohol is the activation of both reward and stress pathways in the brain. The mesocorticolimbic dopaminergic system, involved in reward processing, is activated alongside the corticotropin-releasing factor (CRF)-HPA axis and the autonomic nervous system pathways involved in stress responses. Activation of these central pathways results in increased levels of ACTH and cortisol, as well as changes in heart rate, blood pressure, and skin conductance responses.^10^ Withdrawal and abstinence following chronic alcohol use also are associated with dysfunctional sympathetic and parasympathetic responses, highlighting the effect of alcohol misuse on these peripheral stress pathways; as shown in Figure 2, there are sex differences in these alcohol-related adaptations of the stress pathways.

**Figure 3:**
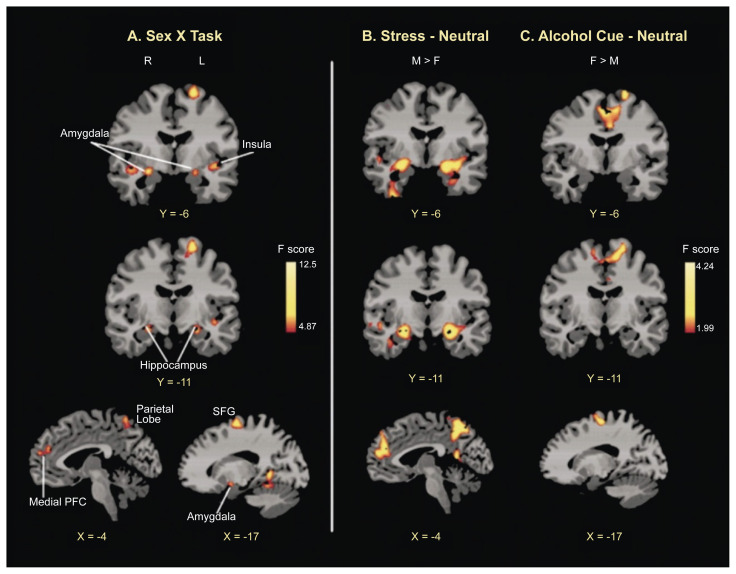
Whole-brain voxel-based functional magnetic resonance imaging (fMRI) showing a sex × condition interaction and corresponding activations in the stress-neutral and alcohol cue-neutral contrasts for males (M) and females (F) who drink socially. **A**: The sex × condition interaction effect was significant in regions of the superior and middle frontal gyrus (SFG/MFG), medial prefrontal cortex (mPFC, dorsomedial and ventromedial), rostral anterior cingulate cortex, emotion limbic regions (posterior insula, putamen, amygdala, hippocampus, and parahippocampal gyrus), temporal lobe, and visuomotor perception areas (parietal lobe, occipital lobe, and cerebellum) (*p* < 0.01 whole-brain familywise error [FWE] rate corrected). To elucidate the source of the interaction, male versus female contrasts were conducted for (**B**) stress relative to neutral, and (**C**) alcohol cue relative to neutral brain responses at the *p* < .05 whole-brain FWE corrected. Significantly, greater M > F stress-induced activity in the mPFC and limbic regions was observed. Alcohol cue-induced activity in the SFG/MFG was significantly higher in women than in men. No differences in F > M for the stress-neutral and in M > F contrast for the alcohol cue-neutral survived whole-brain correction. Coordinates are given in Montreal Neurological Institute space. *Note:* F, female; L, left; M, male; mPFC, medial prefrontal cortex; R, right. *Source:* Reproduced with permission from Seo et al., 2011.^49^ Copyright © 2010 Wiley-Liss, Inc. All rights reserved.

**Figure 4:**
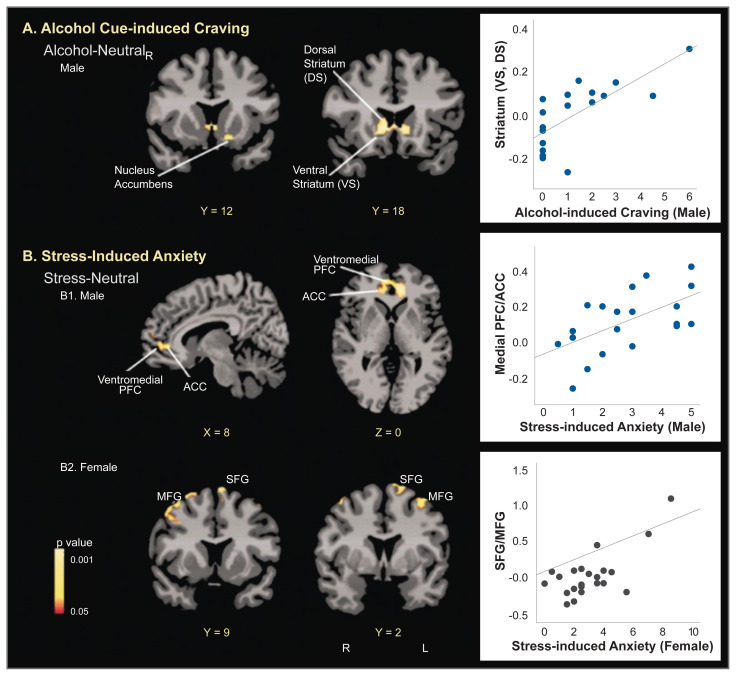
In men and women who drink socially, whole brain voxel-based correlation and corresponding scatter plots for (A) alcohol cue-induced craving ratings with neural responses during alcohol cue versus neutral cue exposure in males as well as (B) stress-induced anxiety ratings with neural response during stress versus neutral cue exposure in males and females (*p* < .05, whole-brain familywise error rate [FWE] corrected). **A**: In males, elevated alcohol craving ratings were associated with increased activity in the striatum cluster (*r* = .74) that encompassed ventral and dorsal striatum, including the left nucleus accumbens (X = −13, Y = 12, Z = −12). **B1**: In males, enhanced stress-induced anxiety ratings were associated with increased brain activity in a medial prefrontal cortex cluster that included the ACC, ventromedial PFC, and medial PFC (*r* = .59). **B2**: In females, stress-induced anxiety ratings were positively correlated with bilateral brain activity in superior/middle frontal gyrus (winsorized *r* = 0.62). Coordinates are given in Montreal Neurological Institute space. *Note:* ACC, anterior cingulated cortex; L, left; MFG, middle frontal gyrus; PFC, prefrontal cortex; R, right; SFG, superior frontal gyrus. *Source:* Reproduced with permission from Seo et al., 2011.^49^ Copyright © 2010 Wiley-Liss, Inc. All rights reserved.

Even though acute administration of drugs, such as alcohol, may increase mesolimbic dopamine levels, sustained alcohol misuse downregulates the mesolimbic dopamine pathways and thus decreases basal dopamine levels.^10^ Using brain imaging, research has shown that there are fewer dopamine D2 receptors and less dopamine transmission in frontal regions and in the ventral striatum area of individuals with AUD during withdrawal.^10^ Furthermore, dopamine response to drugs is sex-specific, with men showing greater dopamine release than women.^47^ Prolonged exposure to drugs, such as alcohol, results in altered and blunted neurochemical responses to drugs as well as to stress. Behavioral sensitization to drugs and stress can also be observed and is associated with CRF and noradrenergic effects on dopaminergic (and non-dopaminergic) pathways and with synaptic alterations in the ventral tegmental area, amygdala, nucleus accumbens, and mPFC.^10^ More importantly, sex differences in both stress and reward circuitry have been reported using functional magnetic resonance imaging (fMRI) research, where responses to stress and to alcohol cues relative to neutral cues show a differential profile in men who drink socially versus women who drink socially^48^ (see Figure 3). Furthermore, although striatal activation during alcohol cue exposure was associated with alcohol craving, this effect was seen in men only and not in women, and different prefrontal regions were associated with stress-induced anxiety in men and women (see Figure 4). These data suggest that central brain pathways differentially modulate stress and alcohol motivation responses in men and women who drink socially and point to a significant need to understand the neurobiology of binge drinking and chronic alcohol misuse in women.

## STRESS NEUROCIRCUITRY, EMOTION REGULATION, AND ALCOHOL CRAVING

Previous human research indicates that trauma, adversity, and chronic stress alter the activity and structure of the prefrontal cortical, limbic, and striatal brain networks involved in regulating stress and emotions as well as reward and higher cognitive or executive control functions.^10^ These brain circuits also show significant sexual dimorphism, suggesting a need to explore the role of sex differences in their structure and function in critical regulation and coping functions for stress, trauma, and self-control over alcohol intake. These functions can include the regulation of distress and emotions, such as controlling and inhibiting impulses, refocusing and shifting attention, employing working memory, monitoring conflict and behavior, linking behaviors to possible future consequences, and demonstrating flexible consideration of alternatives for response selection and decision-making.^10^

**Figure 5:**
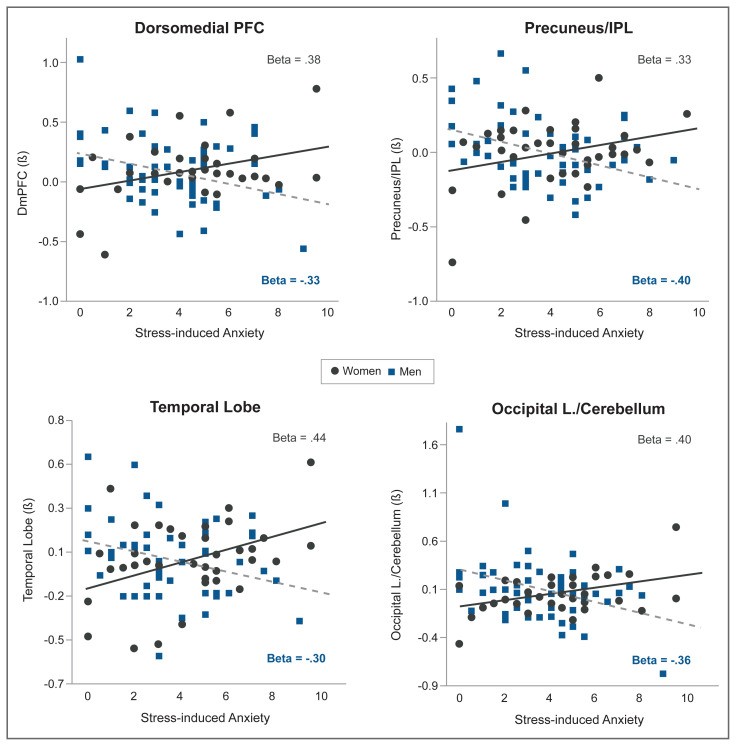
Scatter plots and regression lines for stress-induced anxiety ratings with neural responses during stress relative to neutral-relaxing exposure for specific regions of interest (ROIs). Simple effects in ROIs from whole-brain regression of significant regions from the gender-by-anxiety interaction effects analyses are shown separately in men and women. Stress-induced anxiety predicted brain responses to stress differentially by gender. The plots show (A) positive (women [W]) and negative (men [M]) associations between stress-induced anxiety ratings and activity in the dorsomedial prefrontal cortex (PFC) (W: β = .38; M: β = −.33), precuneus and inferior parietal lobe (W: β = .33; M: β = −.40), middle/inferior temporal gyrus (W: β = .44; M: β = −.30), and occipital lobe and cerebellum (W: β = .40; M: β = −.36). Beta (β) indicates the standardized coefficient. There were no outliers in any of these brain regions for both men and women. *Note:* DmPFC, dorsomedial prefrontal cortex; IPL, inferior parietal lobe; Occipital L., occipital lobe. *Source:* Reproduced with permission from Seo et al., 2017.^48^ Copyright © 1999–2020 Wiley-Liss, Inc. All rights reserved.

Recent evidence from human brain structural and magnetic resonance imaging shows that recent life stressors (e.g., death in family, divorce, relationships ending, being assaulted, financial crises, robberies), trauma (physical, emotional, or sexual abuse), and chronic stress (subjective experience of continual stressors or ongoing life problems) are associated with lower gray matter volume in medial prefrontal, amygdala, hippocampus, and insula regions of the brain.^50^,^51^ Similarly, recent life stress and acute stress exposure (such as those listed above) may decrease responses in the prefrontal regions (such as the dorsolateral prefrontal cortex and ventromedial prefrontal cortex) associated with working memory, reward processing, and resilient coping.^52^ Such changes in the neural circuits underlying emotion and reward dysregulation may promote risky alcohol use (e.g., binge drinking), emotional eating, and frequency of arguments and fights.^52^ Furthermore, these circuits are sexually dimorphic in their responses to stress and anxiety, where differential brain regions are associated with stress-induced anxiety in men versus women^52^ (see Figure 5). As anxiety and stress responses are associated with alcohol motivation and increased alcohol use, sex differences in the neurocircuits that respond to and regulate stress and anxiety suggest that there are also sex differences in the brain regions that drive stress-induced alcohol craving and intake. However, there is a need for examining this association in a sex-specific manner in future research.

Across at-risk children and adults with exposure to stress, trauma, or in utero substance use, sex-specific brain changes in emotion and reward regions are associated with risk of alcohol misuse and AUD.^53^ A study of prenatally cocaine-exposed and non-exposed adolescents (ages 14 to 17) found lower gray matter volume in limbic and frontal regions of the brain as assessed by MRI and whole-brain voxel-based morphometry in the at-risk prenatally exposed relative to non–cocaine-exposed adolescent controls.^53^ In addition, lower gray matter volume in these brain regions was associated with initiation of tobacco, alcohol, and cannabis use.^53^ Furthermore, sex-specific effects were found in adults who misuse cocaine and alcohol, with women showing lower gray matter volume in emotional-limbic regions of the insula, amygdala, and hippocampus, and men showing lower gray matter volume in the midcingulate and frontal regions.^54^ These data suggest that changes in brain volume may serve as biological risk markers for alcohol misuse, AUD, and substance use. Indeed, low behavioral and cognitive control are linked to lower prefrontal and insular cortex volume, and high activation of limbic-emotional and striatal-motivation brain regions under stress suggest one specific pattern underlying risk of addictive behaviors where there is a decreased ability to control rewarding behaviors.^10^ Thus, cortico-striatal reward and motivational brain pathways appear to be key targets of disrupted central stress and emotional responses, suggesting a potentially important sex-specific mechanism by which stress may affect susceptibility to alcohol misuse and AUD vulnerability. As these pathways are sex-specific, the stress- and alcohol-related adaptations also occur in a sex-specific manner, resulting in sex differences in the biological pathways of risk for AUD. However, there is a desperate need for research to elucidate these sex-specific changes and risk factors for AUD.

## TRANSITION TO ADDICTION

Women report different motives for alcohol use than men,^10^,^11^ and are more likely to self-medicate their emotional distress, negative affect stemming from high stress, and mood and anxiety disorders.^10^,^11^ As outlined above, sex differences in addiction vulnerabilities set women at a disadvantage related to exposure to and risk of alcohol misuse, maintenance, and relapse.^11^ As described in the previous sections, some research has documented sex-based differences in neuroendocrine stress and reward pathways with chronic alcohol use.^11^

The cross-sensitization process of stress and alcohol effects suggests that sex-specific adaptations occur with alcohol misuse and chronic use, which may contribute to alcohol craving, continued use, and relapse. The progression from alcohol misuse to AUD often includes overpowering cravings seen as a physiological need rather than a hedonic desire.^10^ This craving is associated with compulsive seeking of alcohol, which becomes stronger in the context of alcohol cues or stress exposure, increasing the chances of relapse. Sex differences in stress assessment and cue reactivity in social drinkers and in patients with AUD have been reported. For example, findings in social drinkers indicate that the incentive value of alcohol may be less sensitized by negative mood and stress in female social drinkers compared with male social drinkers.^55^,^56^ However, findings show that, compared to men with AUD, women with AUD demonstrate greater alcohol cue reactivity following negative mood induction.^57^ Furthermore, HPA-axis hyporeactivity to social stress, alcohol cue exposure, and alcohol intake, as well as a blunted cortisol response to stress in women with AUD have been reported concurrently with enhanced emotional distress and greater craving, which, in turn, have been shown to increase the risk of relapse and return to alcohol use in early treatment.^11^ Although conducted using separate stress- and cue-reactivity paradigms, this research consistently reflects robust sex-specific dissociations between participants with and without AUD in relation to stress system function and alcohol cue reactivity, supporting the notion that there are sex differences in the mechanisms that drive the transition to AUD, its maintenance, and the relapse to alcohol use. However, the specific link between the robust sex-specific stress and cue reactivity responses and actual binge and heavy alcohol intake in women are not clear and needs greater study in future research.

## IMPLICATIONS FOR ONSET AND MAINTENANCE OF AUD IN WOMEN AND FUTURE DIRECTIONS

Sex differences in the onset of alcohol misuse and the development of AUD have been reported. The effects of greater exposure to and experience of stress, trauma, victimization, negative affect, and mood and anxiety disorders in women represent a specific risk pathway for the onset and development of AUD in women. However, estimation bias in occurrence of mood and anxiety disorders needs specific consideration in assessing these associations to alcohol misuse and AUD. Also, although this paper has not focused on genetic mechanisms and epidemiological and sociocultural factors that may explain sex differences, these areas also need further attention. Nonetheless, sex differences in the psychological and biological response to both stress and alcohol intake are well known. Animal studies have revealed that sex steroid hormones interact with the HPA axis to influence stress regulation, and these sex hormones also modulate brain limbic, striatal, and frontal circuits to influence alcohol seeking in sex-specific ways.^11^ However, research in humans assessing interactions between stress, reward, and sex steroid hormones has lagged behind. For example, fluctuations in sex hormones across the menstrual cycle may impact neuroadaptations in stress response and alcohol craving^11^ as described below, and, in doing so, may point to specific prevention and treatment efforts.

Although not specifically examined in risk of AUD or in women with AUD, some evidence in other substance use disorders indicates that during the follicular phase of the menstrual cycle, positive rewarding drug effects may be potentiated in women to the same levels as men.^11^ Similarly, increased levels of progesterone and decreased estrogen/progesterone ratio have been shown in women who misuse substances relative to healthy controls.^11^ Such changes across the menstrual cycle may then alter brain responses to stress and cues as well as affect intensity of emotional responses and craving states in women with AUD relative to men with AUD.^11^ As the hypothalamic-pituitary gonadal (HPG) axis modulates sex steroid levels during the menstrual cycle and influences stress responses in women, adaptations in the HPG and HPA axes with the transition to AUD may lead to altered levels of estrogen, progesterone, and their related neuroactive steroids. This could further predispose women to increased anxiety, negative emotion, and lowered tolerance to stress, which in turn may increase vulnerability to craving and compulsive alcohol use in women.

At a time when alcohol misuse is on the rise among girls, and binge drinking and AUD rates have substantially increased in women, there is a major gap in understanding the mechanisms and processes that specifically increase risks for the onset and development of AUD in girls and women and for the maintenance of AUD in women. Greater specific, targeted future research on risk pathways for girls and women can address the need for focused development of targeted prevention and early treatment efforts in females. Prevention and early treatment may reduce the prevalence rates of AUD—as well as the much higher rates of alcohol-related health problems and morbidity in women compared to men—and such efforts may increase alcohol recovery rates among women.
